# Game-Based Assessment of Spatial Cognition Across a Wide Age Range

**DOI:** 10.3390/bs16040607

**Published:** 2026-04-19

**Authors:** Daniela E. Aguilar Ramirez, Zitong Wu, Catalina Basualto San Martin, Robbin Gibb, Claudia L. R. Gonzalez

**Affiliations:** 1 Department of Kinesiology and Physical Education, University of Lethbridge, 4401 University Drive, Lethbridge, AB T1K 3M4, Canada; zitong.wu@uleth.ca (Z.W.); catalina.basualto.08@gmail.com (C.B.S.M.); claudia.gonzalez@uleth.ca (C.L.R.G.); 2 Department of Neuroscience, University of Lethbridge, 4401 University Drive, Lethbridge, AB T1K 3M4, Canada; gibb@uleth.ca

**Keywords:** game-based measures, spatial cognition, gender differences, age differences, lifespan assessment

## Abstract

Challenges remain in developing a comprehensive understanding of spatial cognition, including gender and developmental differences, partly due to limitations of well-established spatial measures. Many traditional tasks face accessibility constraints and are not well suited for use across broad age ranges, populations, or ability levels. The present study introduced two game-based tasks, Q-bitz^®^ and Spot it!^®^, designed to assess mental rotation and object location memory, respectively. We examined whether these game-based measures meaningfully complement established spatial tests, the Mental Rotation Test (MRT) and the Object Location Memory (OLM) task, across a wide age range (7–79 years, *N* = 114). Results indicated that MRT scores were strongly related to Q-bitz performance, whereas OLM scores were strongly related to Spot it! performance, supporting the convergent validity of the game-based tasks. Notably, gender-specific patterns emerged in the relationships among spatial measures, suggesting differences in spatial function. Age was associated with performance on speeded tasks (Q-bitz and Spot it!) but not with accuracy-based MRT or OLM performance. Together, these findings demonstrate that game-based assessments capture meaningful spatial constructs and reveal gender-specific patterns across the lifespan, providing a practical and ecologically valid approach for advancing research on spatial cognition.

## 1. Introduction

Spatial abilities refer to the capacity to perceive, encode, remember, and mentally manipulate spatial relationships among objects and within environments. These abilities support everyday activities such as fitting furniture through a doorway, locating one’s car in a parking lot, or navigating an alternative route when a familiar street is closed. Beyond their role in daily functioning, spatial abilities are closely linked to important life outcomes and cognitive health. In childhood, early spatial abilities are strong predictors of educational attainment and professional success, particularly in science, technology, engineering, and mathematics (STEM) fields (Shea et al., 2001; Wai et al., 2009; Verdine et al., 2017; Tian et al., 2023). Importantly, these abilities also play a central role in the visual and textile arts (Goldsmith et al., 2016; Bennett-Pierre & Gunderson, 2023; Kus & Newcombe, 2025). Accordingly, there is growing recognition of the importance of integrating the arts into science and technology education (i.e., STEAM), demonstrating the broader relevance of spatial cognition across both technical and creative disciplines. Across adulthood, spatial abilities remain critical: they are among the first cognitive functions to show age-related decline, and impairments in spatial processing can serve as early markers of cognitive disorders, including dementia (Iachini et al., 2009; Techentin et al., 2014). Crucially, across the lifespan and regardless of gender, spatial abilities are malleable (Uttal et al., 2013; Lowrie et al., 2021). This malleability presents a golden opportunity: spatial abilities can be strengthened early in childhood to support STEAM learning, and they can be maintained or improved throughout life to promote cognitive health and everyday functioning. Despite their importance, the developmental trajectories of spatial abilities from childhood to older adulthood, as well as our ability to track individual differences, remain incompletely understood. One contributing factor is the lack of assessments that measure spatial abilities accurately and comprehensively, using measures that are theoretically grounded, accessible, and applicable across age groups, ability levels, and populations. Such assessments are essential for understanding how spatial abilities function, how they develop and change with age, and how they vary across individuals.

Spatial cognition is not a unitary construct but comprises multiple distinct and interacting components. A framework proposed by Uttal et al. (2013) and further developed by Newcombe and Shipley (2014) organizes spatial skills along two key dimensions: intrinsic versus extrinsic spatial information, and static versus dynamic processing demands. This 2 × 2 typology yields four categories of spatial skills and provides a useful structure for classifying spatial tasks and understanding their underlying cognitive demands. For example, intrinsic–static tasks involve processing the configuration of objects (e.g., categorizing shapes based on their spatial features), whereas intrinsic–dynamic tasks require transforming object representations (e.g., mental rotation). In contrast, extrinsic–static tasks involve representing spatial relationships between objects (e.g., object location or map-based tasks), while extrinsic–dynamic tasks require transforming spatial relations between objects, such as during perspective taking. Importantly, different spatial measures may engage different neural mechanisms and may show different patterns of variability across individuals.

### 1.1. Spatial Measures, Assessment Challenges, and Gender Differences

Over more than a century of research on spatial abilities, hundreds of measures have been developed. Two well-studied and widely used measures are the Mental Rotation Test (MRT; Shepard & Metzler, 1971) and the Object Location Memory test (OLM; Silverman & Eals, 1992). The MRT assesses the ability to mentally rotate three-dimensional objects, a core component of spatial reasoning that predicts success in STEM fields and other spatially demanding professions. The OLM requires participants to remember the locations of objects in a visual array, tapping spatial memory and visual search processes relevant for navigation and everyday object tracking. Within the 2 × 2 framework of spatial skills (Uttal et al., 2013; Newcombe & Shipley, 2014), the MRT is typically classified as an intrinsic–dynamic task, as it requires the mental transformation of object representations. In contrast, the OLM is best characterized as an extrinsic–static task, as it involves encoding and recalling the spatial relationships between objects within a layout. The MRT and OLM are valuable tests because they are theoretically grounded and extensively validated. However, despite their value, limitations in these and other spatial measures have hindered progress toward a comprehensive understanding of spatial function, individual differences, and the development of a coherent theoretical framework (Uttal et al., 2024). Uttal et al. (2024) highlight several persistent challenges in spatial assessment. First, many measures suffer from limited accessibility, with some tests difficult to obtain or costly to administer. Second, many tests exhibit age- and population-specific constraints. For example, the MRT is extremely difficult for children and older adults (Hoyek et al., 2012; Jansen & Heil, 2009; Jansen et al., 2013; Aguilar Ramirez et al., 2021, 2022). As a result, the suitability of many spatial tests is reduced when applied to diverse age groups and populations. Third, although individual differences are observed in some tasks, they are not consistent across measures, suggesting that different tasks may tap distinct cognitive processes. Fourth, there is often limited clarity regarding what individual tests measure, and finally, the relationships among different spatial abilities remain largely unexplored. Collectively, these limitations emphasize the need for accessible, adaptable, and theoretically grounded assessments that can complement established benchmarks such as the MRT and OLM, while clearly specifying what each test measures and how these tests may be related.

These challenges in spatial assessment are compounded by the presence of robust gender differences, which underscore the need to understand how different tasks capture distinct spatial processes in males and females. Gender differences in spatial abilities are a well-established and theoretically important phenomenon (Castro-Alonso & Jansen, 2019). Males generally outperform females on mental rotation tasks such as the MRT, whereas females often outperform males on tasks like the OLM, which requires remembering the locations of items in a visual array. These patterns are robust across age groups and cultures (Voyer et al., 1995, 2007; Lippa et al., 2010; Levine et al., 2016; Lauer et al., 2019; Aguilar Ramirez et al., 2021, 2022) and provide a trustworthy benchmark for interpreting spatial performance. Importantly, these gender differences also highlight a broader challenge identified by Uttal et al. (2024): performance on different spatial tasks can vary depending on which cognitive processes are being measured, and some measures detect gender differences while others do not. This inconsistency underscores the need for a clearer theoretical understanding of how spatial abilities and spatial tests are related. Exploring the relationships between new, accessible tasks and traditional benchmarks such as the MRT and OLM is theoretically important because it can help clarify whether mental rotation and spatial memory abilities rely on shared cognitive processes that may also diverge in gender-specific ways. Considering these gender differences and task relationships is essential not only for the development of new assessments, but also for understanding male and female spatial functioning.

### 1.2. Novel Tasks and Study Objectives

To address these gaps, the present study introduces Q-bitz^®^ and Spot it!^®^ as accessible, ecologically valid tasks that may recruit spatial processes overlapping with those engaged by the canonical laboratory MRT and OLM. Importantly, Q-bitz can be situated within a long tradition of visuospatial construction and disembedding tasks. The reconstruction of patterned designs from colored cubes closely parallels the logic of the embedded figures paradigm and resembles the block design tasks used in early split-brain research (Bogen & Gazzaniga, 1965; Sperry et al., 1969). In these classic tasks, successful performance depends on the ability to parse complex visual configurations into component parts and reorganize them into a target whole, a process often described as disembedding and linked to right-hemisphere visuospatial specialization (Bogen & Gazzaniga, 1965; Sperry et al., 1969). Thus, beyond simple pattern matching, Q-bitz is theorized to engage a constellation of spatial transformation processes, including mental rotation, configural analysis, and visuospatial working memory, which overlap with those assessed by traditional measures such as the MRT. In contrast, Spot it! requires the detection of visual items in an array, engaging visual search and selective attention processes, and may also involve aspects of spatial comparison relevant to object–location processing. These tasks are not assumed to be pure measures of single spatial constructs. Rather, they are conceptualized as multi-component tasks that may overlap with processes assessed by traditional measures such as the MRT and OLM. Both tasks are inexpensive, widely available, and suitable for administration across childhood to older adulthood, making them practical for research spanning diverse populations. Importantly, these tasks allow us to examine how performance on novel, accessible measures relate to established spatial tests. Although their conceptual alignment with the MRT and OLM is not yet established, using Q-bitz and Spot It! offers an opportunity to examine accessible, lifespan-appropriate alternatives. This approach also allows us to test whether these game-based measures can meaningfully complement traditional benchmarks in spatial cognition research.

While specific hypotheses were tested, the present study also includes an exploratory component aimed at examining how these novel, game-based tasks relate to established spatial measures, given that their precise construct alignment has not yet been fully established. Given the Q-bitz and Spot it! characteristics we hypothesized that Q-bitz performance would significantly predict MRT scores, and Spot it! performance would significantly predict OLM scores. Based on established findings with traditional measures, we further hypothesized that males would outperform females on the MRT and Q-bitz, whereas females would outperform males on the OLM and Spot it! tasks. Finally, the wide age range of our sample allowed us to investigate age-related changes in performance on these tasks, providing insight into how spatial and game-based measures may differentially capture developmental and aging-related effects across the lifespan.

## 2. Method

### 2.1. Participants

**Figure 1:**
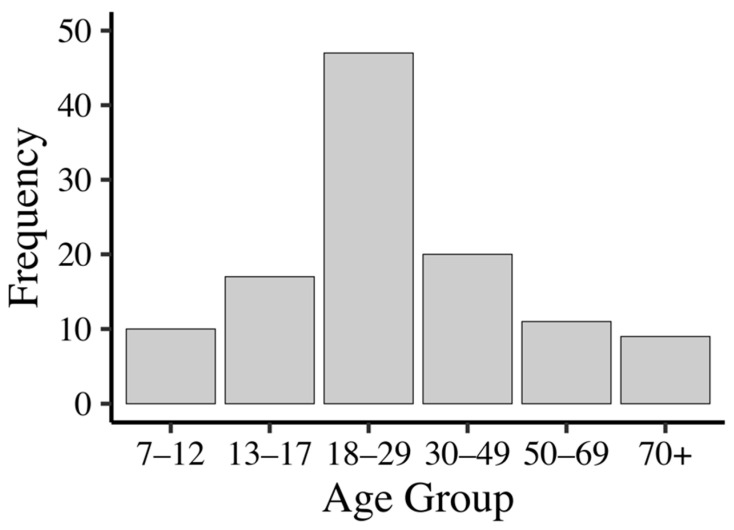
Distribution of participant ages across the sample. Bars represent frequency within age bins.

A hundred and fourteen participants (64 females) took part in the study from a wide age range (7–79 years old, M = 30.33, SD = 18.78). To provide a clearer characterization of the sample, the overall age distribution is illustrated in Figure 1 and the distribution of participants across age groups and by gender is presented in Table 1. Although participants spanned a wide age range, representation across age groups was not uniform. Note, the purpose of the study was not to investigate differences in spatial ability across discrete age groups, but rather to capture variability across a broad age range in order to enhance the generalizability of the findings. Consistent with this aim, age was modeled as a continuous variable in all analyses, allowing us to account for age-related variance without imposing arbitrary group boundaries or reducing statistical power.

All participants were healthy, without any diagnosed neurological conditions. The sample was recruited in southern Alberta and broadly reflected the demographics of the region. Participants primarily identified as Caucasian (*n* = 65, 57%), followed by Asian (*n* = 24, 21%), Latino (*n* = 10, 9%), mixed race (*n* = 9, 8%), and African American (*n* = 6, 5%).

Participants were recruited through word-of-mouth within the Lethbridge community, the University of Lethbridge news platforms, or were recruited through the University of Lethbridge Department of Psychology’s participant management system (Sona Systems) and received course credit for their participation. Written informed consent was obtained from all adult participants. For participants under the age of 18, a parent or legal guardian provided written consent. Additionally, participants aged 15–17 years who demonstrated decision-making capacity provided their own assent/consent; capacity was assessed using a conversation-based approach evaluating the participant’s ability to understand the study, appreciate its potential risks and benefits, reason about participation, and communicate a voluntary choice. The study was approved by the University of Alberta Research Ethics Board 2.

### 2.2. Tasks

#### 2.2.1. MRT (Mental Rotation Test)

**Figure 2:**
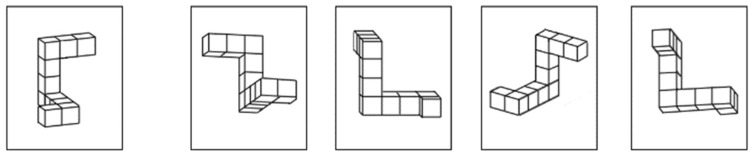
The Mental Rotation Test. Note. This example illustrates one item from the Mental Rotation Test (MRT). The shape on the left is the target, and it is paired with four comparison shapes on the right. Participants must identify which two of these comparison shapes represent rotated versions of the target. In the example shown, the correct choices are the second and third figures.

The Mental Rotation Test (MRT) included two blocks of 12 items. For each item, a target shape appeared on the left side of the display, accompanied by four comparison shapes on the right (Figure 2). Participants had to identify which two of the four comparison figures were the correct rotated versions of the target (Peters et al., 1995).

#### 2.2.2. OLM (Object Location Memory Test)

**Figure 3:**
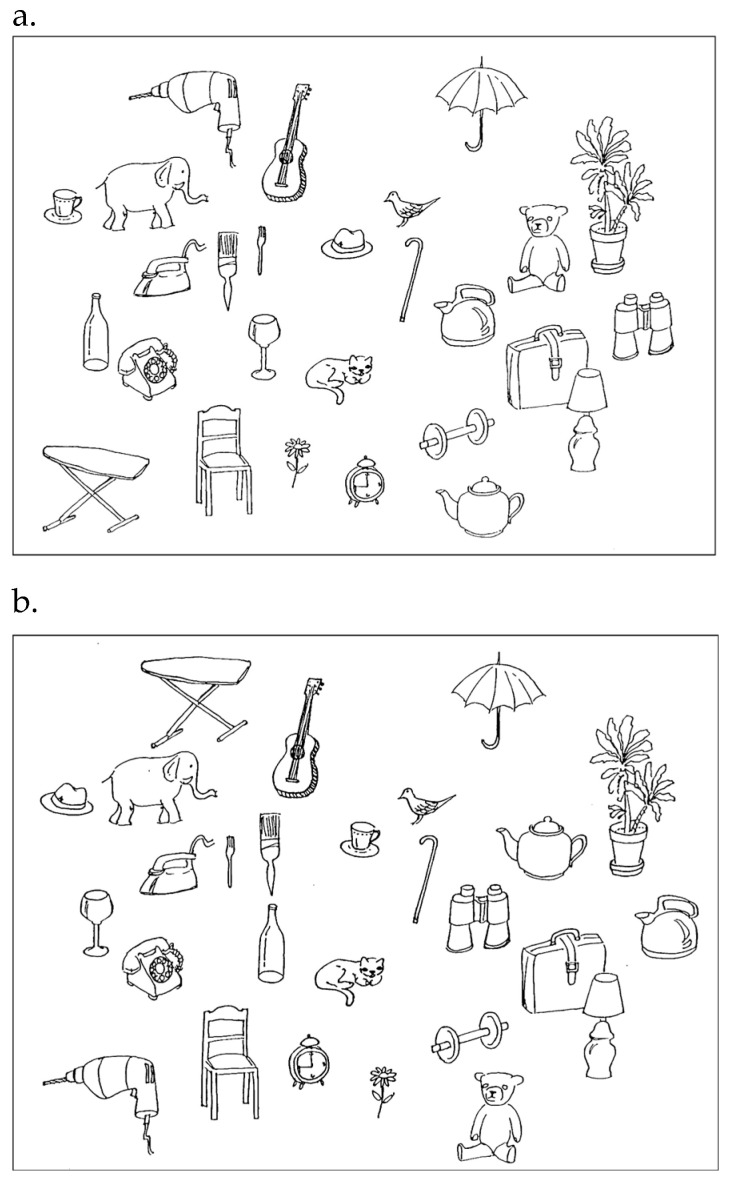
The Object Location Memory Test. Note. (**a**) First sheet shown to participants during the 60 s study phase. (**b**) Second sheet in which participants identified objects that had changed location by circling them.

Participants viewed a sheet of objects for 60 s and were asked to memorize their locations (Figure 3a). They then saw a second sheet in which some objects had been moved and were asked to circle the ones that had changed position. Participants had 60 s to circle the items (Figure 3b; adapted from Silverman et al., 2007; Silverman & Eals, 1992).

#### 2.2.3. Q-Bitz

**Figure 4:**
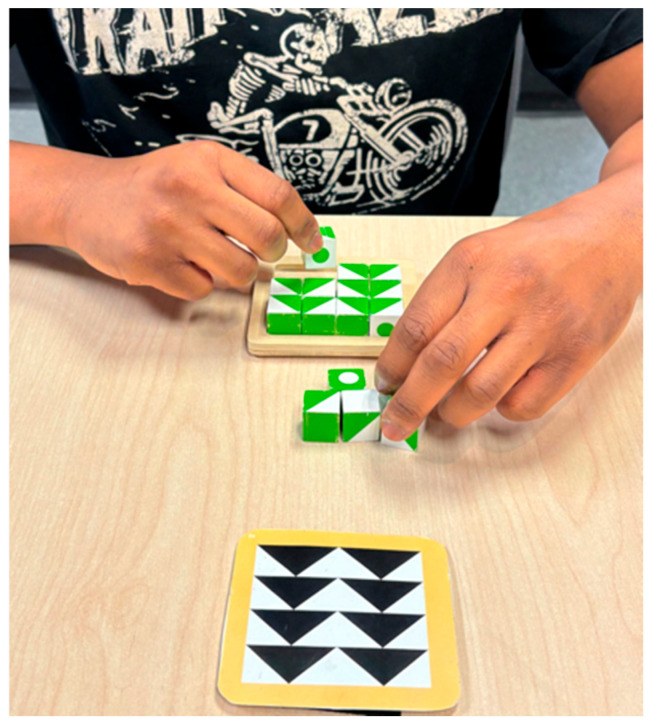
Q-bitz Task.

In the Q-bitz task, participants were required to reproduce a target pattern using the wooden cubes provided (Figure 4). Each participant completed six trials.

#### 2.2.4. Spot It!

**Figure 5:**
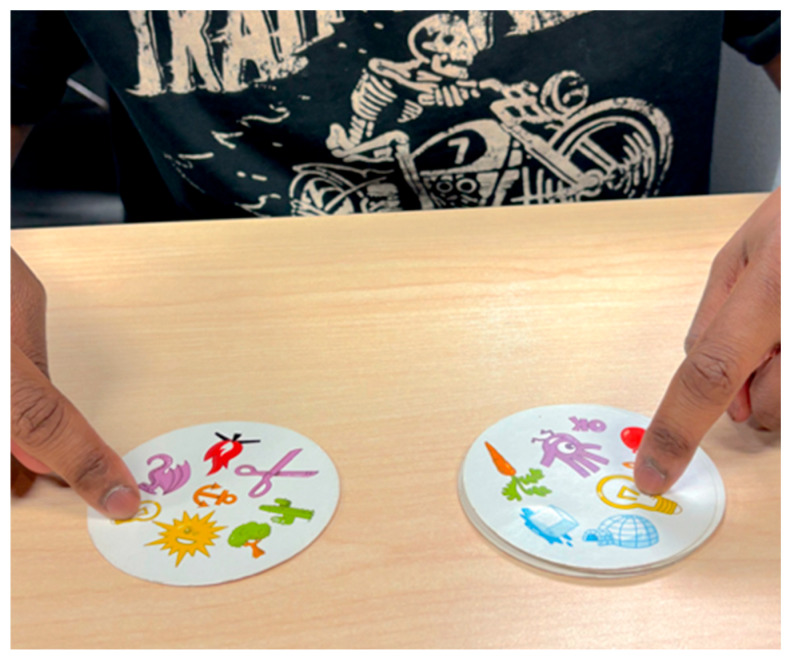
Spot it! Task.

The Spot it! task involved participants identifying the single matching symbol shared between two cards presented to them (Figure 5). All fifty-five cards from the game were used.

### 2.3. Procedure

Before taking part in the study, participants or parents/caregivers were asked to review and sign an informed consent/assent form, as well as complete a personal information form. The personal information form gathered basic demographic details; participants self-reported their age, or the age of their child, gender, and handedness.

All tasks (MRT, OLM, Q-bitz, and Spot it!) were counterbalanced in order of presentation. For the MRT, participants were verbally instructed to identify the two figures, from a set of four options, that matched the target figure. Each block of 12 items was timed, with participants given three minutes to complete each block, and a three-minute rest interval was provided between blocks.

In the OLM task, participants first studied the arrangement of objects on an initial sheet for 60 s, with instructions to memorize their locations. After the 60 s, the sheet was removed and participants were presented with a second sheet in which some objects had been moved and asked to circle the objects they believed had changed position within 60 s.

For the Q-bitz task, participants were shown a target pattern for each trial and instructed to replicate it using wooden cubes as quickly and accurately as possible. The cubes were shuffled at the start of each trial, and the order of trials was counterbalanced across participants.

In the Spot it! task, participants were asked to identify the single matching symbol shared between two cards. Upon verbally indicating the match, the cards were placed on top of each other and a new card was presented alongside. The deck was shuffled three times before each participant.

### 2.4. Data Analysis

The MRT was scored as the percentage of correct responses, calculated by summing only the trials in which both answers were correct, dividing by the maximum score of 24, and multiplying by 100 (Peters et al., 1995).

OLM performance was calculated by adding the number of correct selections (i.e., those that moved) and subtracting the number of incorrect selections (i.e., those that did not move; Silverman et al., 2007).

For Q-bitz, the time for each trial was measured from the experimenter’s “go” signal until the participant completed replicating the pattern. The mean completion time across the six trials was computed for each participant. To assess the reliability of the Q-bitz task, internal consistency across trials was evaluated using Cronbach’s alpha.

In the Spot it! task, the total time was recorded from the presentation of the first two cards until the participant correctly identified the matching symbol of the last card.

Given the wide age range of the sample (7–79 years) and evidence that age-related changes in cognitive performance are often non-linear across the lifespan (Hartshorne & Germine, 2015; LaPlume et al., 2021), age was modeled using both linear and quadratic terms. Age was mean-centered prior to analysis (Age_c = Age − sample mean age), and a quadratic term (Age_c^2^) was derived from the centered age variable. This approach permitted the detection of non-linear age–performance relationships, reduced multicollinearity between age terms, and allowed age effects to be interpreted relative to the average participant.

Multiple linear regression analyses were conducted to examine possible relationships between the well-established MRT and OLM tests and the introduced Q-bitz and Spot it! tasks. Given our main hypothesis, in one model, MRT served as the dependent variable, with Qbitz, OLM, Spot it!, and age_c and age_c^2^ as predictors (entered simultaneously). In a second model, OLM was the dependent variable, with Q-bitz, Spot it!, MRT, age_c and age_c^2^ as predictors (entered simultaneously). Furthermore, to further explore any effects of gender, these two multiple regression models were then performed the same but with Gender included as an interaction term in both models. Assumptions for linear regression were evaluated for all models (MRT and OLM as dependent variables). Linearity and homoscedasticity were assessed using visual inspection of residuals versus predicted value plots, and normality of residuals was examined using histograms of standardized residuals. Multicollinearity was assessed using variance inflation factors (VIF).

A one-way multivariate analysis of covariance (MANCOVA) was conducted to examine gender differences across the spatial tasks. The dependent variables were MRT, OLM, Q-bitz and Spot it!, with centered age and centered age squared included as covariates. Assumptions for MANCOVA were evaluated. Visual inspection of residual histograms and Q–Q plots indicated that residuals were approximately normally distributed, although formal tests of normality were significant for some variables. Given the sensitivity of these tests to sample size, these deviations were not considered substantial. Levene’s tests indicated that the assumption of homogeneity of variance was met for most dependent variables, with the exception of MRT. Box’s M test was significant, indicating a violation of the assumption of equality of covariance matrices. Given the robustness of Pillai’s Trace to such violations, multivariate effects were interpreted using Pillai’s Trace. Significant multivariate effects were followed by univariate ANCOVAs to examine task-specific gender differences while controlling for age. An alpha level of 0.05 was applied for all statistical comparisons. IBM SPSS Statistics (Version 29) was used for all analyses.

## 3. Results

### 3.1. Psychometric Evaluation and Model Assumption Testing of Game-Based Spatial Measures

Inspection of residual plots indicated that the assumptions of linearity were met for both regression models (MRT and OLM as dependent variables). For the MRT model, some variability in residual spread was observed at higher predicted values; however, this did not indicate a severe violation of homoscedasticity. For the OLM model, residuals were evenly distributed across predicted values, supporting the assumption of homoscedasticity. In both models, residuals were approximately normally distributed, with only minor deviations from normality. Variance inflation factors (VIF) were equal to 1.0 for all predictors, indicating no evidence of multicollinearity.

The Q-bitz task demonstrated good internal consistency across trials (Cronbach’s α = 0.87, 95% CI [0.83, 0.90]), indicating reliable performance across repeated trials. As the Spot it! task yielded a single aggregate performance measure (total completion time), traditional reliability indices could not be computed.

To assess validity, we examined the relationships between the game-based tasks and established spatial measures. Q-bitz performance was a significant predictor of MRT performance, and Spot it! performance significantly predicted OLM performance, providing evidence of convergent validity.

### 3.2. Relationships Among Spatial Tasks: Overall and Gender-Stratified Regression Analyses

A multiple linear regression analysis (enter method) was conducted to examine if any of the relevant variables predicted performance on MRT. Thus, MRT was entered as the dependent variable and performance on the OLM, Q-bitz, Spot it! plus Age c, and Age_c^2^ as predictors. The model was significant (*F*_(5, 106)_ = 7.76, *p* < 0.001, *R*^2^ = 0.27), with the Q-bitz being the only predictor of performance. This relationship was negative, the higher the score on the MRT, the lower the time in the Q-bitz (see Table 2).

A similar analysis was conducted to explore significant predictors of OLM performance. Thus, OLM was entered as the dependent variable and performance on the MRT, Q-bitz, Spot it! plus Age c, and Age_c^2^ as predictors. The model was significant (*F*_(5, 106)_ = 3.99, *p* = 0.002, *R*^2^ = 0.16) with performance on Spot it! as the only predictor of OLM scores. This relationship was negative, the higher their score (better performance) on the OLM, the lower the time on Spot it! (see Table 2). Note that age centered or quadratic did not emerge as significant predictors in any of the analyses.

To further explore any effects of gender, additional analyses were conducted with gender included as an interaction term in both models.

**Figure 6:**
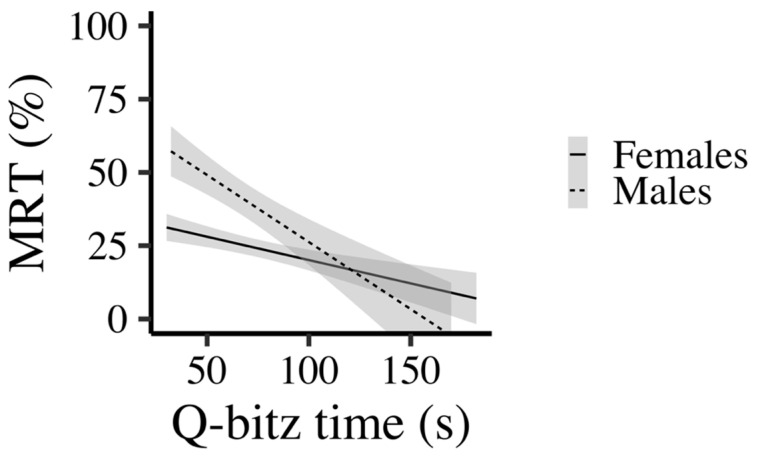
Interaction between Gender and Q-bitz performance.

For the MRT model, there was a significant interaction between Q-bitz and gender (β = −0.58, *p* = 0.006), indicating that the relationship between Q-bitz performance and mental rotation differed by gender. Specifically, the negative slope was stronger for males when compared to females; the higher their score on the MRT, the lower the time in the Q-bitz (see Figure 6). Importantly, gender also showed a strong main effect (β = 0.81, *p* = 0.002), with males exhibiting higher MRT scores overall. Together, these findings suggest a male-specific link between Q-bitz performance and mental rotation.

For the OLM model, the interaction (Spot-it time × Gender) did not reach significance (*p* = 0.19), indicating that the relationships between OLM and Spot it! were consistent across genders. Additionally, gender itself was not a significant predictor of OLM performance (*p* = 0.53). Overall, these results suggest that, unlike MRT, object location memory does not exhibit gender-specific associations with the game-based measures.

Note that previous research has demonstrated that the MRT is particularly challenging at the extremes of the lifespan, including in children (especially those younger than 13 years) and in older adults (Linn & Petersen, 1985; Hoyek et al., 2012; Frick & Möhring, 2013; Jansen & Heil, 2009; Aguilar Ramirez et al., 2021, 2022). Similarly, performance on object location memory tasks may be sensitive to task demands in younger samples, with prior studies modifying the paradigm (e.g., reducing the number of items) to better accommodate children (Barnfield, 1999).

Accordingly, to evaluate whether the observed relationships were influenced by potential floor effects at the lower end of the age range, additional regression analyses were conducted in restricted samples excluding younger participants (13–58 years and 18–58 years). The pattern of results remained consistent across both subsamples, with Q-bitz performance significantly predicting MRT performance and Spot it! performance significantly predicting OLM performance. These findings indicate that the observed associations were not driven by performance limitations in younger or older participants.

### 3.3. Spatial Tasks Gender and Age Differences

Assumption checks indicated no substantial violations of linearity or normality. While Levene’s test was significant for MRT and Box’s M test indicated unequal covariance matrices, results were interpreted using Pillai’s Trace, which is robust to such violations.

**Figure 7:**
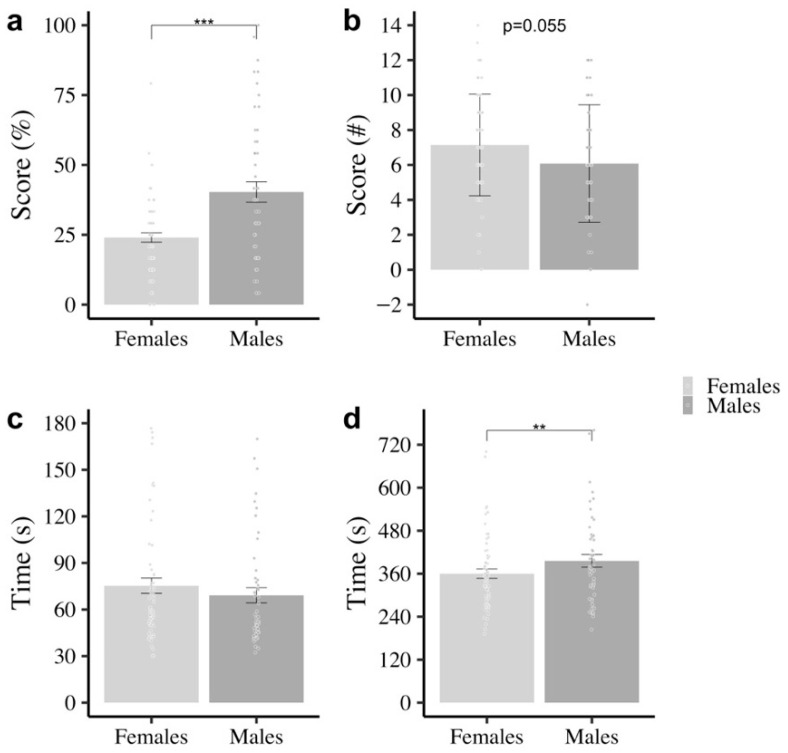
Performance of female and male participants in the tasks. (**a**) MRT; (**b**) OLM; (**c**) Q-bitz, and (**d**) Spot it! tasks. Note. *p* ≤ 0.01 **, *p* ≤ 0.001 ***. Error bars represent standard errors.

A MANCOVA was conducted to examine gender differences in the MRT, OLM, Q-bitz and Spot it! tasks, controlling for age using both linear (mean-centered) and quadratic age terms to account for potential non-linear age-related effects. After adjusting for age, there was a significant multivariate effect of gender, Pillai’s Trace = 0.27, *F*_(4, 105)_ = 9.79, *p* < 0.001, $\eta_{p}^{2}$ = 0.27. Follow-up univariate ANCOVAs revealed a significant gender difference in the MRT, *F*_(1, 108)_ = 15.52, *p* < 0.001, $\eta_{p}^{2}$ = 0.13, and in Spot it! *F*_(1, 108)_ = 6.01, *p* < 0.05, $\eta_{p}^{2}$ = 0.05. Males scored higher (better) than females on the MRT, but females were faster at completing the Spot it! task than males. A marginal gender difference was found on the OLM *F*_(1, 108)_ = 3.76, *p* = 0.055, $\eta_{p}^{2}$ = 0.03. Females scored higher than males at the task. No gender differences were found in the Q-bitz task (*p* > 0.1; see Figure 7).

Furthermore, age centered squared (Age_c^2^) was a significant covariate in the model, Pillai’s Trace = 0.11, *F*_(4, 105)_ = 3.23, *p* < 0.05, $\eta_{p}^{2}$ = 0.11. Follow-up univariate ANCOVAs indicated that age quadratic (age_c^2^) was strongly associated with Q-bitz *F*_(1, 108)_ = 12.66, *p* < 0.01, $\eta_{p}^{2}$ = 0.11 and Spot it! completion times *F*_(1, 108)_ = 5.50, *p* < 0.05, $\eta_{p}^{2}$ = 0.05. Importantly, these effects reflect a nonlinear relationship, suggesting that performance changes across the lifespan are not uniform but instead vary at different stages of development and aging. However, age quadratic was not significantly related to MRT scores or OLM scores (*p*’s > 0.1). Note, the age linear term was not a significant covariate of the model.

## 4. Discussion

Achieving an integrated understanding of spatial cognition that accounts for individual differences and development remains a central challenge in cognitive neuroscience. Despite the substantial value of widely used spatial measures, limitations in these and other tasks have constrained progress toward this goal. In particular, differences in task demands, age suitability, and spatial construct clarity have made it difficult to compare findings across studies and populations. Collectively, these challenges highlight the need for spatial assessments that are both theoretically grounded and adaptable across differences in abilities and developmental stages. Such measures should complement established benchmarks, while clearly specifying the spatial constructs they assess and how they relate to one another. The present study was designed to address this need by introducing game-based spatial tasks that target mental rotation and object location memory and are suitable for use across a wide age range. By examining their relationships with established measures, as well as gender and age differences, the study aims to contribute to creating a more comprehensive understanding of spatial cognition.

Consistent with our first hypothesis, performance on the game-based tasks was significantly associated with performance on established spatial measures. Specifically, Mental Rotation Test (MRT; Shepard & Metzler, 1971) performance was significantly predicted by Q-bitz performance, and Object Location Memory (OLM; Silverman & Eals, 1992) performance was significantly predicted by performance on the Spot it! task. These associations provide preliminary evidence of convergent validity of the game-based measures, indicating they engage spatial processes that overlap with those assessed by the MRT and OLM across a wide range of ages and ability levels. Importantly, these tasks are not considered direct or pure measures of the same constructs, but rather multi-component tasks that share underlying spatial processing demands with established measures. Although age was included as a predictor in both regression models, it did not account for the majority of explained variance. Instead, Q-bitz performance emerged as the strongest predictor of MRT performance, and Spot it! performance emerged as the strongest predictor of OLM performance. This pattern suggests that shared spatial processing demands across tasks contribute uniquely to performance beyond age-related influences. Together, these findings support the suitability of Q-bitz and Spot it! as accessible, low-cost spatial assessments that can be used across age groups, facilitating meaningful comparisons throughout development.

An important consideration in interpreting these findings is that the tasks differ in their primary dependent measures. Whereas MRT and OLM rely on accuracy-based outcomes, Q-bitz and Spot it! are based on completion time, which may reflect both spatial processing and general processing speed. Accordingly, the observed relationships between tasks should be interpreted as reflecting overlapping cognitive demands rather than direct equivalence in underlying constructs. Future research would benefit from incorporating measures that separately assess speed and accuracy, allowing for a clearer distinction between spatial ability and general processing speed.

### 4.1. Gender Differences in Spatial Performance and Task Relationships

When gender was included as an interaction term in the regression models, MRT performance was best explained by Q-bitz scores, but only in males. In contrast, OLM scores did not interact with gender, indicating that the relationship between OLM and the game-based task Spot it! was consistent across females and males. These results suggest that gender differences in task relationships are process-specific, emerging most clearly for mental rotation. In males, the association between MRT and Q-bitz indicates that performance on tasks placing similar demands on spatial transformation is more closely aligned, even when these tasks differ in format. Notably, this relationship spans task format, with MRT requiring the mental rotation of objects presented on a two-dimensional surface and Q-bitz involving the construction of three-dimensional configurations. This convergence is consistent with the dimensionality-crossing hypothesis (Horan & Rosser, 1984), which posits that two-dimensional representations of three-dimensional objects must first be transformed into a three-dimensional mental representation prior to rotation. The observed MRT–Q-bitz relationship in males suggests that this additional representational step does not disrupt alignment across tasks, allowing performance to generalize across formats.

In contrast, the absence of a comparable relationship between the MRT and Q-bitz in females suggests that they may not rely on the same underlying mechanisms, and/or that female performance may be more sensitive to differences in task format. Prior work indicates that females are more influenced by the mode of stimulus presentation and may adopt more effective strategies when depth information is directly available (Parsons et al., 2004; Neubauer et al., 2010). Moreover, females have shown greater difficulty with paper-based spatial tasks in which a three-dimensional structure must be inferred from incomplete or occluded two-dimensional representations (Voyer & Doyle, 2010), as is the case in the MRT. Within this framework, the lack of an MRT–Q-bitz relationship in females may reflect greater sensitivity to dimensionality-crossing demands, such that performance on tasks requiring transformation from two-dimensional to three-dimensional representations does not align as closely with performance on tasks where three-dimensional structure is directly available.

Our second hypothesis was partially supported; gender differences in spatial ability were found, but not in all the tasks. As hypothesized, males outperformed females in the MRT, and an approaching significant difference (*p* = 0.055) was found on the OLM, with females scoring higher than males at the task. These findings are consistent and replicate the large effect size found on the MRT (e.g., Voyer et al., 1995; Levine et al., 2016) and the small to moderate effect size found on the OLM (Voyer et al., 2007). Contrary to our hypothesis, however, males did not outperform females on the Q-bitz task. This may be explained by a couple of reasons. First, this result is consistent with previous research showing that the large gender difference typically observed on the MRT is diminished or even eliminated when rotation stimuli are presented in more ecologically valid or three-dimensional (real-world) formats (e.g., Robert & Chevrier, 2003; Hawes et al., 2015; Aguilar Ramirez et al., 2020; Larson et al., 1999; Parsons et al., 2004; Neubauer et al., 2010). As discussed above, these effects are thought to reflect reduced dimensionality-crossing demands when depth information is directly available. Second, evidence suggests that females and males may employ different strategies when solving spatial tasks, with females more likely to adopt a piecemeal or analytic approach and males more likely to rely on holistic processing (Pletzer, 2014). Therefore, we suggest the structural demands of assembling individual cubes into a coherent whole may attenuate gender differences by favoring analytic strategies more commonly used by females. Future studies incorporating process-level measures, such as eye-tracking or neuroimaging techniques, may help elucidate these task-specific strategy differences. Regardless of the reason, the findings raise the possibility that Q-bitz may provide a more inclusive assessment of mental rotation ability by reducing structural demands that may differentially influence performance on the MRT.

### 4.2. Age-Related Effects Across the Lifespan

Age-related effects in the present study were best captured by a quadratic function rather than a linear trend, consistent with prior evidence demonstrating that developmental trajectories of cognitive abilities, including spatial cognition, are non-linear across the lifespan (Hartshorne & Germine, 2015; LaPlume et al., 2021). Specifically, age centered squared was significantly associated with Q-bitz and Spot it! completion times, but not with accuracy on the MRT or OLM. This pattern suggests that age-related changes in performance were specific to tasks emphasizing speed, rather than tasks that rely on accuracy. This pattern may also reflect broader age-related changes in general processing speed, rather than differences in spatial ability per se, and therefore should be interpreted with caution when attributing these effects solely to spatial cognition. This interpretation aligns with previous findings showing that processing speed tends to exhibit earlier and steeper age-related changes than accuracy-based performance across cognitive domains (Sharps & Gollin, 1987; LaPlume et al., 2021; Rahe & Quaiser-Pohl, 2023).

Notably, the absence of significant age effects on MRT and OLM accuracy may not necessarily be evidence that performance on these tasks remains stable across the lifespan. Rather, it is more likely that both tasks posed substantial difficulty at the extremes of the age distribution (i.e., in children and older adults), thereby attenuating observable age-related effects. Previous research has demonstrated that the MRT, in particular, is highly challenging for both children and older adults (Frick & Möhring, 2013; Hoyek et al., 2012; Aguilar Ramirez et al., 2021, 2022; Jansen & Heil, 2009). Similarly, the same OLM task was administered across all age groups without any age adjustments, this may have imposed constraints on performance for younger and older participants. Consistent with this interpretation, in the current study mean accuracy was relatively low (approximately 32% for MRT and 48% for OLM), suggesting the presence of floor effects that may have obscured underlying age-related differences in these tasks. Importantly, in the regression analyses (which included both linear and quadratic age terms), the relationships between game-based and traditional measures remained consistent when analyses were restricted to age ranges in which the MRT and OLM are more appropriate (13–58 or 18–58), suggesting that the observed associations were not driven by floor effects in younger or older participants. Furthermore, the heterogeneity of the sample across a wide age range may have further obscured more fine-grained developmental trends. Future studies using larger, age-stratified samples or more flexible modeling approaches (e.g., spline regression) may better capture developmental trajectories in spatial cognition.

### 4.3. Limitations and Conclusion

The current study has some limitations that warrant consideration. First, reliance on reaction time as the primary outcome measure for the Q-bitz and Spot it! tasks may have limited the ability to examine relationships between speed and accuracy across tasks, as well as to fully characterize gender and age-related performance. Second, the MRT and OLM tasks and procedures were not modified for younger children or older adults. Although this decision was made to maintain methodological consistency across ages, task difficulty may have constrained performance at the extremes of the lifespan, thereby obscuring potential age-related associations. Third, the Spot it! task yielded a single aggregate performance measure (total completion time), which precluded the calculation of reliability indices. As a result, the reliability of this measure could not be formally established within the current study. Future research should incorporate repeated administrations to enable a more comprehensive evaluation of the reliability of this task. Fourth, socioeconomic status (SES) was not collected, limiting the ability to examine how SES factors may influence spatial cognition and task performance. Given that SES has shown to influence spatial cognition and its gender differences (Bower et al., 2020; Levine et al., 2005), future studies should incorporate SES measures to better characterize the sample and its potential influence on performance. Finally, although the study aimed to include participants across a wide age range, developmental changes in these abilities may have been masked by task difficulty and uneven age representation. Accordingly, future studies could employ these game-based tasks within sufficiently powered, age-stratified samples to better characterize gender and developmental trajectories across the lifespan.

In conclusion, the present study used game-based ecologically valid assessments to examine spatial processes related to mental rotation and object location memory. Rather than treating spatial ability as a unitary skill, the tasks were selected and evaluated based on their alignment with well-defined spatial processes and their suitability for use across a wide age range. The findings demonstrate that engaging and scalable measures can capture established spatial abilities. By emphasizing construct clarity alongside adaptability across differences in ability and age, this work highlights the value of theoretically grounded, game-based assessments for studying individual differences, developmental trajectories, and the organization of spatial function.

## Figures and Tables

**Table 1:** Distribution of participants across age group by gender.

Age Group	Total (*N*)	Females (*n*)	Males (*n*)
7–12	10	5	5
13–17	17	9	8
18–29	47	27	20
30–49	20	11	9
50–69	12	7	5
70+	8	5	3

**Table 2:** Results of the regression analyses. Only significant predictors are shown for each regression model.

Dependent Variable	Significant Predictor	*B*	SE	β	*t*	*p*
MRT	Q-bitz	−0.25	0.06	−0.42	−4.07	<0.001
OLM	Spot it!	−0.01	0.003	−0.27	−2.52	=0.01

## Data Availability

The materials and data that support the findings of this study are available through Mendeley Data (Mendeley Data, V1, doi: 10.17632/mfsc9b3czd.1).
